# Silver Splits and Parent–Child Disconnectedness: Mental Health Consequences for European Older Adults

**DOI:** 10.1007/s10680-025-09751-9

**Published:** 2025-10-14

**Authors:** Lisa Jessee, Deborah Carr

**Affiliations:** 1 Department of Sociology and Social Psychology, University of Cologne, Albertus-Magnus-Platz, 50923 Cologne, Germany; 2 Department of Sociology and Center for Innovation in Social Science, Boston University, 704 Commonwealth Ave., Boston, MA 02215 USA

**Keywords:** Gray divorce, Depressive symptoms, Parent–child relationships, Intergenerational solidarity, Union dissolution, Well-being

## Abstract

Rising rates of “silver splits” in Europe resemble increases in gray divorce in the U.S. Partnership dissolutions may harm older adults’ mental health, especially for ‘disconnected’ parents who do not receive support from their children. However, researchers have relied primarily on multilevel modeling, neglecting unobserved characteristics that may select an individual into both divorce and parent–child disconnectedness. This brief report addresses this research gap by estimating fixed-effects linear regression models that control for time-invariant confounders. We used data from the Survey of Health, Ageing and Retirement in Europe (SHARE; 2004–2022, *N* = 2216 observations, 546 silver splits) to document changes in depressive symptoms pre- and post-dissolution and evaluate whether these patterns are moderated by parent–child disconnectedness. Consistent with previous research, we find that depressive symptoms increase steeply in the year of dissolution and remain high four years post-dissolution for parents who are disconnected from their adult child(ren). However, individuals who maintain a relationship with all their child(ren) show stable levels of depressive symptoms throughout the dissolution process, challenging the assumption that dissolution is uniformly distressing. Our results reveal that depressive symptoms trajectories during the period preceding and following a major life event differ across sociorelational contexts. Social programs and supports for divorced older adults should recognize this heterogeneity rather than assuming uniformly negative mental health outcomes.

## Introduction

Rates of ‘silver splits’—the dissolution of marriages or romantic partnerships at or after age 50—have risen in Europe, mirroring ‘gray divorce’ trends in the U.S. (Alderotti et al., 2022; Brown & Lin, 2022; Solaz, 2021; Vignoli et al., 2025; Žilinčíková & Schnor, 2021). The number of relationship dissolutions in later life is expected to rise further in the coming years, given population aging (Brown & Lin, 2012). The loss of a co-residential intimate partner through relationship dissolution may deprive adults of a crucial source of socioemotional and instrumental support (Brown & Lin, 2022). Union dissolutions may be especially consequential for older adults because age-related transitions including retirement, onset of health problems, and the deaths of peers may diminish their levels of contact with other potentially supportive ties (Charles & Carstensen, 2002).

Classic writings on stressful life events emphasize that transitions like divorce may trigger mental health symptoms including depression, due to both the acute stress of the event and the sequelae of secondary stressors that follow, such as a drop in household income or the loss of a helpmate and confidante (e.g., Norris & Murrell, 1987). The divorce-stress-adjustment model counters that union dissolution is a process that can affect mental health even *before* the event due to precipitating stressors like persistent relationship conflict and the anticipation of future disruptions to daily life and everyday routines (Amato, 2000).

Empirical assessments of the divorce-stress-adjustment framework draw on three distinctive conceptual models which offer competing perspectives regarding the time course of mental health symptoms *following* later-life dissolution (Lin & Brown, 2020). The *crisis model* proposes that union dissolutions lead to short-term mental health declines with a relatively quick recovery as individuals manage temporary stressors like legal issues or residential changes, whereas the *chronic strain model* predicts persistent mental health struggles from ongoing stressors, such as loneliness and the challenges of managing household chores and finances independently (Lin et al., 2019).The *convalescence model*, specific to older adults, similarly predicts long-lasting mental health decrements yet an eventual improvement due to older adults’ resilience and capacity to adapt in the longer term (Lin & Brown, 2020; Lin et al., 2024).

Yet, the extent to which mental health trajectories during the silver split process align with the crisis, chronic strain, or convalescence models may be contingent on the availability of coping resources—most notably protective social relationships (e.g., Pearlin et al., 2005). For older adults experiencing union dissolution, a strong relationship with adult children is considered an important buffer against emotional distress (Tosi & van den Broek, 2020). Conversely, strained, conflictual or tenuous parent–child relationships may intensify and prolong the emotional consequences of dissolution. Recent research identifies one particular dimension of weak parent–child ties that heightens the mental health effects of dissolution: *parent–child disconnectedness* (Jessee & Carr, 2025; Kalmijn, 2023; Lin et al., 2024). Disconnectedness is similar to estrangement, which encompasses deficient emotional closeness and frequency of contact, and refers to the parent’s lack of contact with at least one adult child (Agllias, 2018; Arránz Becker & Hank, 2022; Conti, 2015; Reczek et al., 2023; Scharp & Hall, 2017). An emphasis on disconnectedness from *at least one child* is consistent with research documenting that parental well-being is affected even when difficulties or strains exist with only a single child (Fingerman et al., 2012; Reczek et al., 2025). In Europe, disconnectedness affects between 1 and 17% of the population, with divorced men (13%) experiencing it roughly twice as often as divorced women (6%; Jessee & Carr, 2025).

Disconnectedness may exacerbate and lengthen older adults’ post-dissolution mental health symptoms because disconnected parents may be deprived of practical or emotional support from adult child(ren), a resource critical to their adaptation (Lin et al., 2024). Moreover, disconnectedness may be a co-occurring stressor, creating a *double burden* that may further erode the mental health of older adults experiencing a silver split. Disconnectedness is more consequential than strong positive parent–child relations for older adults’ mental health because weak ties are atypical, stigmatized, and emotionally painful (Gilligan et al., 2015; Lin et al., 2024). Older parents who are disconnected from their adult children may experience a more significant and prolonged increase in mental health symptoms during a silver split compared to connected parents. This scenario is consistent with the chronic strain perspective, as opposed to the crisis or convalescence perspectives suggested by previous research on the mental health effects of gray divorces (Lin et al., 2019; Tosi & van den Broek, 2020). Thus, our aim is to evaluate the extent to which depressive symptoms trajectories pre- and post-divorce differ on the basis of parent–child disconnectedness. We focus specifically on depressive symptoms because this is one of the most common mental health problems among older adults. Untreated depressive symptoms also undermine physical health and are projected to become a leading cause of disability worldwide (Andreas et al., 2017; Mathers & Loncar, 2006).

To our knowledge, Lin et al. (2024) are the only researchers who have examined parent–child disconnectedness as a contextual factor affecting depressive symptom trajectories before, during, and after gray divorce. They tracked a sample of US older adults and found that parents reported heightened depressive symptoms post-dissolution, yet the magnitude of this change was significantly larger for those who were disconnected from (vs. connected to) their adult child(ren). The difference between disconnected and connected parents began to diminish two years after the dissolution and converged six years thereafter. However, the authors estimated multilevel models and did not consistently account for unobserved factors that may “select” individuals into later-life separation, depressive symptoms, and disconnectedness.

Moreover, it is unclear whether similar patterns would occur beyond the U.S., given theoretical writings underscoring that the emotional consequences of a purported stressor may be conditional upon the broader sociocultural context (Pearlin et al., 2005). Europe has lower rates of both divorce and parent–child disconnectedness relative to the U.S. (Jessee & Carr, 2025; National Center for Health Statistics, 2023; Reczek et al., 2023), so older adults experiencing the “double burden” of these two stressors may be susceptible to stigmatization, lack of structural supports, and other factors that may render them particularly vulnerable to sustained depressive symptoms.

Against this background, this brief report examines depressive symptoms pre- and post-gray dissolution for two groups of “silver splitters” in Europe: parents who are connected versus disconnected from their adult children. We draw on longitudinal data from eight waves (2004–2022) of the Survey of Health, Ageing and Retirement in Europe (SHARE) and apply fixed-effects regression models. A fixed-effects approach allows us to capture both *observed* and *unobserved* individual confounding factors by subtracting the individual mean from the observed values of all variables.

## Methods

### Data

Data are from SHARE, a longitudinal biennial survey of adults aged 50+ in Europe and Israel (Börsch-Supan et al., 2013), spanning eight waves (1 to 9, excluding wave 3) from 2004 to 2022 across 26 nations. Unlike single-nation surveys, SHARE allows us to follow within-person trajectories among parents who recently experienced a silver split, stratified by parent–child disconnectedness. We excluded Israel (the only non-European country in SHARE) and Ireland (with only one wave of data), as well as wave 3 data, which only included retrospective life course information. Our baseline sample comprises 146,868 individuals, with baseline defined as the first wave in which a respondent is observed in the panel.

Our main goal is to trace *within-person changes* in older parents’ depressive symptoms pre- and post-relationship dissolution. Thus, we limit our sample to parents who are at risk of and subsequently experience a silver split, defined as a marital or non-marital romantic partnership dissolution after age 50 (Alderotti et al., 2022) We exclude from our analytic sample individuals who are: under 50 (*n* = 3740); not in a partnership at baseline (*n* = 44,008); continuously partnered throughout the observation period (*n* = 58,516); respondents with children under the age of 18 or childless (*n* = 6624); with only one wave of data (n = 33,433); or who experienced two or more silver splits during the study (*n* = 1). Respondents with children under the age of 18 were excluded as they were not at risk of experiencing disconnectedness from an adult child. Our sample includes 546 parents who experienced a silver split, with an average of four observations per participant, totaling 2216 observations.

### Measures

*Depressive symptoms* are assessed at each available time point before, during and after separation with the 12-item EURO-D scale (Prince et al., 1999). Respondents indicate whether they have experienced each symptom in the past 12 months: depression, pessimism, suicidality, guilt, sleep, interest, irritability, appetite, fatigue, concentration, lack of enjoyment, and tearfulness. Symptom counts range from 0 to 12. We used a natural log transformation to address the skewed distribution of symptoms (*M* = 2.6, SD = 2.3). Results were consistent across models, so we retain the count for ease of interpretation (results available from authors).

Our focal independent variable is the time to and since a *silver split*. Following previous research (Alderotti et al., 2022; Vignoli et al., 2025), we did not stratify the analyses by dissolution subtype, but instead pooled marital and non-marital transitions into a single category of silver splits. This decision was made for both conceptual and empirical reasons. Our independent variable encompasses persons who either: transitioned from married to divorced (*n* = 472); ended a registered partnership (*n* = 20); or dissolved a cohabiting relationship (*n* = 53) during the observation period. In many European countries, registered partnerships are comparable to marriages, offering some of the same legal rights and benefits (for an overview of how registered partnerships align with or differ from marriage rights across countries, see Scherpe & Hayward, 2017). To have adequately powered moderation analyses, we combined the latter two groups into a single non-marital dissolution category (*n* = 73). Descriptive statistics (person-wave observations) for the two dissolution categories are presented in Table 1. We did not detect statistically significant differences in depressive symptoms (2.70 vs. 2.40) or rates of parent–child disconnectedness (8 vs. 12%) for marital versus non-marital dissolutions. Multivariable analyses showed no significant differences in symptom trajectories between marital and non-marital unions, and restricting our analytic sample to marital dissolutions only resulted in similar patterns as for the full sample of silver splitters (see Appendix Fig. 3). Based on these considerations, we did not differentiate between dissolution subtypes in the analyses and instead proceeded with a pooled analysis of all dissolution subtypes.

**Table 1:** Descriptive statistics by relationship dissolution status

	Marital dissolution	Non-marital dissolution	Difference
Depressive symptoms (range: 0–12)	2.70	2.40	*ns*
	(2.42)	(2.12)	
Parent–child disconnectedness	0.08	0.12	*ns*
Age at baseline	65.13	65.48	*ns*
	(6.50)	(7.23)	
Self-rated health			
Excellent	0.11	0.08	*ns*
Very good	0.22	0.16	*ns*
Good	0.37	0.29	*ns*
Fair	0.21	0.37	***
Poor	0.09	0.10	*ns*
Perceived financial difficulties	0.37	0.41	*ns*
Employed	0.32	0.37	*ns*
Female	0.53	0.51	*ns*
European region			
Northern	0.27	0.27	*ns*
Eastern	0.15	0.31	***
Southern	0.13	0.05	**
Western	0.45	0.37	*ns*
Any values imputed	0.06	0.13	**
Person-wave observations	1,020	153	

Means (and standard deviations) shown for continuous measures and proportions shown for categorical measures. Values based on person-wave data. Statistically significant differences denoted as *p < *.*05, **p < .01, ***p < .001, *ns* not significant. SHARE, waves 1, 2, 4, 5, 6, 7, 8, 9, release 9.0.0. unweighted data

SHARE data lack precise information on divorce/separation dates. Thus, we documented depressive symptoms throughout the dissolution process using five timing indicators based on the current and last interview year. While SHARE’s biennial design typically results in interviews every two years, some respondents are observed one or three years apart due to irregular interview lags. To account for this variability, we grouped years −1 to −3 into a single pre-split category and years +1 to +3 into a single post-split category. We created the following categories: (1) at least four years pre-split (baseline and reference group), (2) between one to three years pre-split, (3) the year in which the split was first recorded, (4) one to three years post-split, and (5) more than four years post-split. In the first (4+ years pre-split) and last (4+ years post-split) categories, we pooled all observations that occurred 4 years or more before or after the split. Consequently, respondents could contribute multiple observations to these categories if they had multiple data points in these time frames. To assess the robustness of this coding strategy, we conducted supplemental analyses in which we truncated the sample to exclude all person-years observed more than four years after the silver split, focusing only on individuals observed exactly four years post-split. The results remained substantively unchanged (results available upon request). Given the increased statistical power afforded by the pooled approach, we retained this specification in our main analyses.

Due to panel attrition and irregular participation across waves, not all individuals contribute observations at every time point before and after the split. For example, a respondent may participate at wave *t*, drop out in *t* + 1, and return in *t* + 2, resulting in missing data for intermediate periods. As a result, individuals contribute varying numbers of observations across the silver split stages. Table 2 displays the distribution of timepoints.

**Table 2:** Number of observations across silver split stages

	*N*	%
Years before/after the silver split		
−4 or more	714	32.2
−1 to 3	302	13.6
0 (year of the silver split)	546	24.6
+1 to 3	204	9.2
+4 or more	450	20.3
Person-wave observations	2216	100

SHARE, waves 1, 2, 4, 5, 6, 7, 8, 9, release 9.0.0. unweighted data

Following Lin et al. (2024), *parent–child disconnectedness* in the year of the split refers to parents’ lack of contact with at least one adult child in the 12 months pre-split (in person, by phone, or mail). Parent–child disconnectedness was included as a time-invariant moderator, measured in the wave in which the silver split was first reported. We chose a time-invariant moderator, since recent simulation studies indicate that interacting two time-varying variables in a fixed-effects model can produce spurious results (Giesselmann & Schmidt-Catran, 2022). Focusing on disconnectedness from at least *one adult child* (as opposed to all children) aligns with research showing that having *any* estranged adult children can negatively affect parental health (Reczek et al., 2025). The reference category includes parents in contact with all of their children. In our analytic sample, 10 percent of persons (49 individuals) were disconnected from at least one adult child and are thus classified as disconnected. While the number of disconnected parents who experience separation in our sample is relatively small, the study provides an initial investigation of this under-researched subgroup, yielding insights that can advance and encourage further research on the heterogeneity of later-life dissolution.

Of the group of disconnected parents, 29 percent were disconnected from all of their children. Depressive symptoms did not differ significantly for the two subgroups of disconnected parents, so we did not stratify analyses by number of children from whom the parent is disconnected. In the year of the silver split, 61 percent of disconnected parents were separated from a biological child, and 39 percent from a stepchild. However, we did not have sufficient statistical power to further stratify our multivariable analyses based on whether the tie was biological. SHARE does not provide information on whether a disconnected child is from the dissolved marriage or partnership or from a prior relationship.

(Un-)observed time-variant confounders, such as number of children, education, genetic factors, personality traits, and relationship duration, are accounted for in our fixed-effects modeling approach. Hence, we adjusted for *time-varying* covariates only. In line with divorce research that applies fixed-effects linear regression models (Kapelle & Monden, 2024; Leopold, 2018), we included a parsimonious set of categorical covariates to capture age and period effects, to account for the possibility that well-being systematically changes with age or varies across historical periods. Age was included as a four-level categorical variable—50–59 (reference), 60–69, 70–79, and 80 and older—and interview year likewise as 2004–2007 (reference), 2010–2013, 2015–2017, and 2019–2022, based on the years in which the data were collected. Including categorical controls avoids the problem of perfect collinearity. Sensitivity analyses using alternative interval lengths and quadric age terms produced very similar results (all models available from authors).

For the 14 percent of respondents who had a missing value on at least one variable used in the analysis, we imputed missing values using chained equations (See Appendix Table 5 for item-specific missingness). We imputed 10 datasets, performed all analyses on each imputed dataset and combined coefficients. Multivariable analyses included a variable signifying *whether any values were imputed.*

To avoid “over‐adjusting” for variables on the causal pathway, we excluded additional time‐varying covariates, including health status, employment status, and financial vulnerability, because these factors may mediate the effect of a silver split on depressive symptoms (Amato, 2000). For example, among women, a silver split may influence depressive symptoms through reduced financial security, whereas among men these effects may operate via diminishing social networks. If we adjust for these mediators as simple covariates, we risk *under*stating the true impact of separation on depressive symptoms. A dedicated mediation analysis would be preferable, but our sample lacks sufficient statistical power (Kohler et al., 2024). Incorporating several time-varying covariates in our model, including employment status (working vs. not working), financial vulnerabilities (financial difficulties vs. no financial difficulties), self-rated health (range: 1 = excellent to 5 = poor), and repartnering (repartnered vs. still separated), yields similar effects, but with slightly reduced magnitude as we anticipated. All covariates were measured at each available SHARE interview wave across the different stages of the silver split.

### Analytic Plan

We apply fixed-effects linear regression models with robust standard errors to account for unobserved characteristics that may confound the associations among silver splits, disconnectedness, and depressive symptoms. Omitting important (unobserved) variables can lead to an over- or underestimation of the true effects (*omitted variable bias*). Fixed-effects panel regression models allow researchers to address omitted variable bias for *unobserved* stable individual characteristics, such as personality traits, childhood experiences, or genetic factors, by subtracting the individual mean from the observed values of all variables (Vaisey & Miles, 2017). Moreover, fixed-effects panel regression models automatically account for *observed* time-invariant characteristics, such as gender, education, and number of children. We find sufficient within-individual variation to justify a fixed-effects approach (see Appendix Table 6).

Fixed-effects regression models use each individual as their own control over time to focus on changes that occur *within* individuals. We modeled a change in depressive symptoms as a function of time before, during, or after the split among (a) connected and (b) disconnected parents. To test for statistically significant differences between connected and disconnected parents, we estimated fully interacted models (see Appendix Table 7 for complete results). The basic equation without interactions is specified as follows:

$${\text{EUROD}}_{\text{it}}= {\beta }_{1} {\text{SILVERSPLIT}}_{\text{it}}+ {\beta }_{2}{x}_{2\text{it}}+\dots + {\beta }_{k}{x}_{\text{kit}}+ {\mu }_{i }+ {\varepsilon }_{\text{it}}$$

where EUROD represents the EURO-D score, our outcome, for individual *i* at time *t*. SILVERSPLIT, our focal predictor, categorizes periods relative to the split event. The other time-varying covariates (age, interview year, and whether values were imputed) are accounted for by variables *x*2, …, *xk* for individual *i* at time *t*. The term *μi* captures individual-specific characteristics that remain constant over time (fixed effects) for individual *i*. Finally, *Ɛ* denotes the error term that varies across both time and individuals (Replication files available here https://osf.io/akc7z/?view_only=811f115e9d7a416890b70e660239af71).

## Results

### Bivariate Analysis

Table 3 provides a statistical portrait of connected and disconnected silver splitters, highlighting the average number of reported depressive symptoms in each subgroup. Disconnected silver splitters report significantly more depressive symptoms than their connected counterparts (3.64 vs. 2.54), suggesting an association between parent–child disconnectedness and increased depressive symptoms in this population.

**Table 3:** Descriptive statistics by parent–child disconnectedness status

	Disconnected	Connected	Difference
Depressive symptoms (range: 0–12)	3.64	2.54	***
	(2.54)	(2.30)	
Age at baseline	63.12	62.27	*ns*
	(7.32)	(7.07)	
Self-rated health			
Excellent	0.10	0.12	*ns*
Very good	0.13	0.23	**
Good	0.32	0.36	*ns*
Fair	0.32	0.21	***
Poor	0.13	0.07	**
Perceived financial difficulties	0.52	0.35	***
Employed	0.28	0.43	***
Female	0.29	0.55	***
European Region			
Northern	0.21	0.26	*ns*
Eastern	0.19	0.18	*ns*
Southern	0.06	0.13	**
Western	0.54	0.43	**
Repartnered post-dissolution	0.01	0.01	*ns*
Any values imputed	0.14	0.11	*ns*
Person-wave observations	211	2005	

Means (and standard deviations) shown for continuous measures, and proportions shown for categorical measures. Values based on person-wave data. Statistically significant differences denoted as *p < .05, **p < .01, ***p < .001, *ns* not significant. SHARE, waves 1, 2, 4, 5, 6, 7, 8, 9, release 9.0.0. unweighted data.

**Fig. 1:**
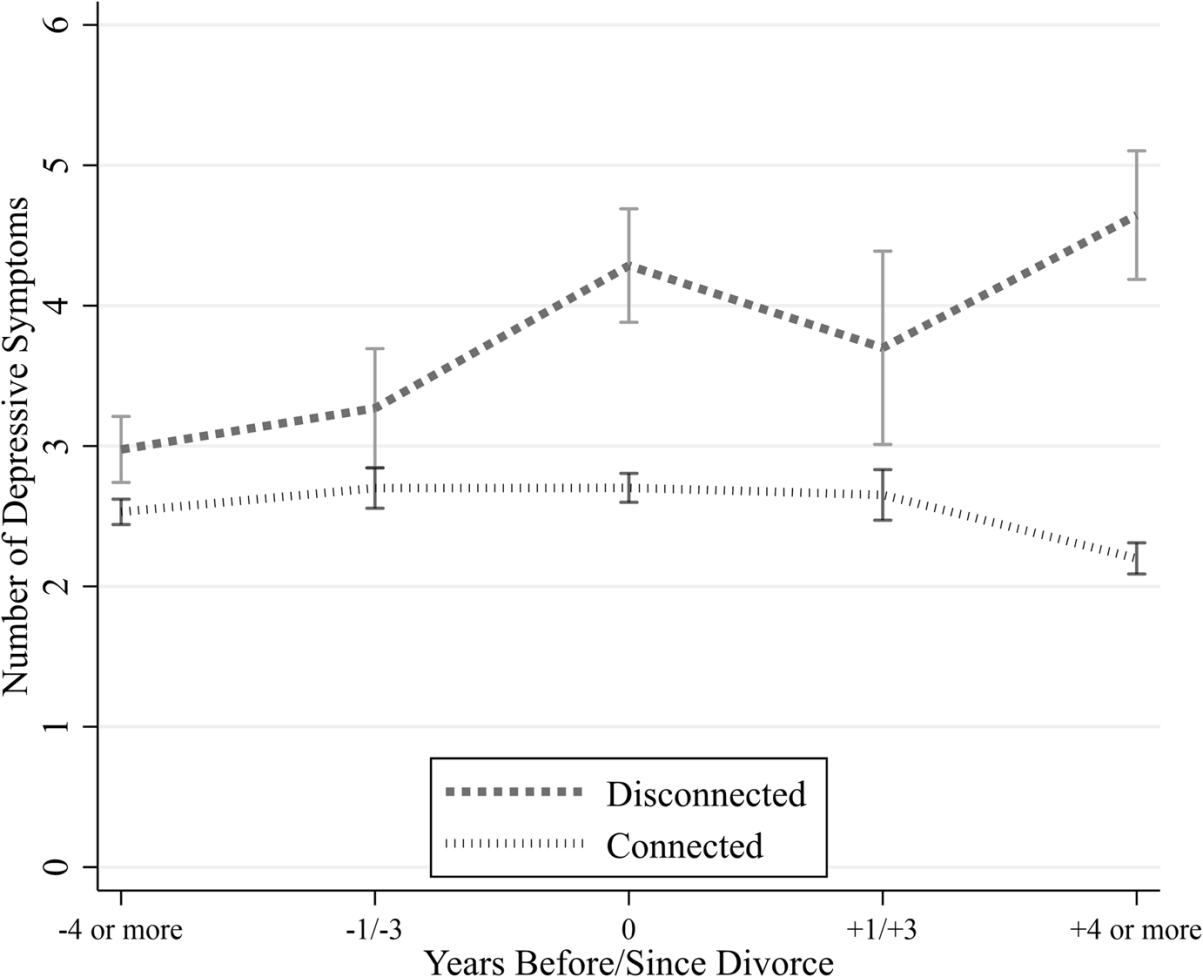
Number of depressive symptoms over time, stratified by parent–child disconnectedness. Bars represent standard deviation. SHARE, waves 1, 2, 4, 5, 6, 7, 8, 9, release 9.0.0. unweighted data

Figure 1 provides a descriptive snapshot of average depressive symptoms at several time points before and after a silver split, separately for connected and disconnected parents. Disconnected parents report higher mean levels of depressive symptoms than their connected counterparts across all observed time points. Moreover, their average symptom levels increase across the observed periods surrounding the silver split. Among connected parents, mean levels of depressive symptoms appear relatively stable across time, with slightly lower values observed four or more years after the separation.

### Multivariable Analysis

**Fig. 2:**
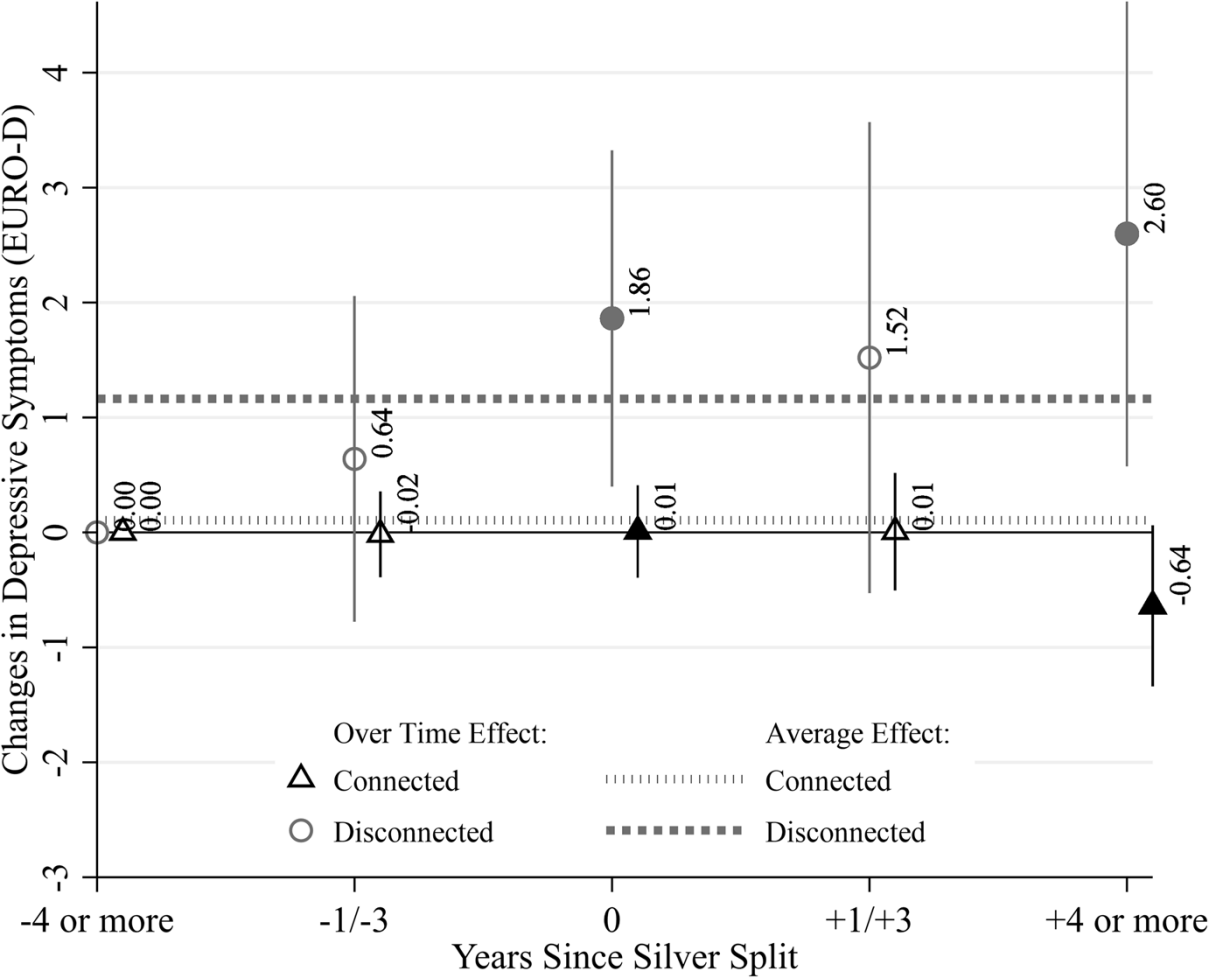
Changes in depressive symptoms during the four years before and after a silver split. Fully adjusted regression coefficients and 95% confidence intervals shown. Solid markers indicate statistically significant differences at *p* < .01 between connected and disconnected respondents. SHARE, waves 1, 2, 4, 5, 6, 7, 8, 9, release 9.0.0. unweighted data

However, the bivariate associations in Table 3 and Fig. 1 may be spurious because they do not account for selection into divorce, so we proceed with longitudinal fixed-effects regressions. These models evaluate the extent to which parent–child disconnectedness is associated with depressive symptoms trajectories, net of time-constant confounders and covariates included in the model. Figure 2 shows depressive symptoms changes across the time period four years pre- to four years post-dissolution for parents who are disconnected versus parents who are connected. Regression coefficients are presented in Table 4.

**Table 4:** Fixed-effects linear regression models predicting changes in depressive symptoms over time

	(1)	(2)
	Disconnected	Connected
	*B*/(SE)	*B*/(SE)
Years since silver split (ref.: −4 or more)	0.00	0.00
	(.)	(.)
−1/−3	0.75	−0.07
	(0.61)	(0.17)
0 (Year of silver split)	2.00**^a^	−0.02^*a*^
	(0.61)	(0.17)
+1/+3	1.70^†^	0.02
	(0.86)	(0.23)
+4 or more	2.53**^a^	−0.52^†* a*^
	(0.94)	(0.30)
Age in categories (ref.: 50–59 years)	0.00	0.00
	(.)	(.)
60–69 years	0.10	−0.11
	(0.50)	(0.16)
70–79 years	0.24	0.08
	(1.01)	(0.30)
80+ years	1.01	0.15
	(1.72)	(0.67)
Interview year in categories (ref.: 2004–2007)	0.00	0.00
	(.)	(.)
2010–2013	−0.95^†^	0.23
	(0.54)	(0.20)
2015–2017	−1.01	0.23
	(0.76)	(0.23)
2019–2022	−2.12*	0.31
	(1.01)	(0.34)
Flag: Imputed values	0.29	−0.07
	(0.71)	(0.18)
Person-wave observations	211	2005
Individual observations	49	497

Statistically significant differences between disconnected and connected parents are denoted with superscripts ^*a*^*p* < .01. SHARE, waves 1, 2, 4, 5, 6, 7, 8, 9, release 9.0.0. unweighted data

^†^*p* < .1, *p < .05, **p < .01, ***p < .001

The circles and triangles indicate levels of *within-person change* in depressive symptoms *over time* for disconnected and connected parents, respectively; solid symbols denote statistically significant differences. The dotted lines denote the *average change* in depressive symptoms after experiencing a dissolution, regardless of the time point during the process. We show this contrast to demonstrate how studies that fail to consider distinctive time points offer an incomplete portrayal of the effects of dissolution on depressive symptoms.

Three main findings emerged. First, disconnected parents experience significant increases in depressive symptoms throughout the dissolution process, although these changes do not unfold in a linear pattern. Depressive symptoms increase slightly before the split, but the change is modest (0.75) and not statistically significant, showing no evidence of anticipation effects. They then experience a significant increase in depressive symptoms in the year of the split (2.00), with this increase persisting and even rising slightly (2.53) four years post-dissolution. In the short term after dissolution (two years), they show some improvement from the transition year but still fare significantly worse than at baseline (1.70). However, this effect is only marginally significant (*p* < 0.10). Although the disconnected group is small, the results suggest that mental health may erode following dissolution and might not return to pre-dissolution levels even four or more years later, consistent with patterns described in chronic strain models.

Second, among connected parents, depressive symptoms showed no significant changes from four years before to two years after a split. However, four or more years post-split, these parents experienced a significant *decrease* in symptoms, reporting 0.52 fewer symptoms compared to baseline. This suggests that connected parents may not experience significant mental health declines during or immediately after a dissolution; rather, they appear relatively resilient and may even show signs of improvement in mental health over time.

Third, disconnected parents consistently show larger changes in depressive symptoms compared to connected parents. However, these disparities reached statistical significance only in the year of the split and at the latest observed time point, as indicated by the significant interaction coefficients (see Appendix Table 7 for interaction results).

## Discussion

Given rising rates of silver splits in the U.S. and Europe (Alderotti et al., 2022; Brown & Lin, 2022; Vignoli et al., 2025), understanding the mental health of older adults who experienced the dissolution of a romantic partnership is a timely goal. Extensive research documents the negative emotional consequences of later-life separation (Hu et al., 2024; Lin et al., 2024; Tosi & van den Broek, 2020), yet this brief report advocates for a more nuanced perspective. We emphasize the importance of considering other risk (or resilience) factors in the face of dissolution, including parent–child (dis)connectedness.

Consistent with previous research (Lin et al., 2024), we found that among the 49 disconnected parents in our analytic sample, depressive symptoms increase steeply in the year of dissolution and remain high for at least four years post-dissolution. However, unlike prior studies focused on the U.S. (Lin et al., 2024), we found that older European adults who maintain relationships with all of their children have relatively low and stable levels of depressive symptoms throughout the dissolution process. Moreover, their depressive symptoms even decrease in the longer term, four years post-dissolution. Thus, while disconnected parents experience silver splits as a chronic strain and experience persistent depressive symptoms, their counterparts who are connected to their children are relatively resilient and even experience improvements in mental health throughout the dissolution process. These results are encouraging, indicating that for older European adults who dissolve their unions and are connected with their children, the depressogenic effects of dissolution are negligible.

Our brief report has focused on a novel contextual factor that moderates the effects of a particular stressful life event (dissolution) on mental health: parent–child (dis)connectedness (e.g., Pearlin et al., 2005). Connected parents in our sample demonstrate resilience and adaptability during the process of later-life dissolution. Support from children may enable them to manage the stress associated with the union dissolution and to seek out or even embrace new opportunities for happiness. Our findings also may reflect the instrumental or material support provided by adult children (Lin et al., 2024); parents who maintain a relationship with all of their children may face less economic insecurity or fewer unmet care needs, mitigating common secondary stressors of dissolution, especially among women (Leopold, 2018; Lin et al., 2019). Connected older parents may even feel sufficiently supported and empowered to dissolve an unhappy union to protect or improve their mental health, particularly because they have a strong support network they can rely on during challenging times. Importantly, our fixed-effects approach also addresses the possibility that unobserved factors like a disagreeable personality or a history of poor-quality relationships contribute to the processes documented here.

However, consistent with work by Lin et al. (2024), our study recognizes the significance of weak parent–child relationships in older adults’ adaptation to the silver split process. Although parent–child disconnectedness is relatively rare, disconnected parents are a subpopulation worthy of scholarly attention, given their potential long-term vulnerability to depression. We encourage health care practitioners and social workers to evaluate not only the number but perceived quality of older adults’ social ties including those ties that are dissolved or strained. This information may guide professionals to work with older adults in identifying or strengthening ties with other family members, friends, neighbors, or professionals who may support in the aftermath of later-life separation.

Whereas our findings broadly align with Lin et al. (2024) in terms of disconnected parents, our results diverge with respect to connected parents, for whom our results suggest *no* increase in depressive symptoms during a silver split. These divergent findings may reflect both methodological (i.e., different methods used) and the broader sociocultural context (Europe vs. U.S.). The use of fixed-effects regression estimation allowed us to account for selection into silver split due to, for instance, personality traits, genetic factors, or the length of the partnership.

Sociocultural factors, including cultural expectations regarding family ties, also may account for the divergent results for the U.S. and European-based studies. For instance, cross-national research by Silverstein et al. (2010) has shown that the relationship between parents and their adult children in the U.S. is more likely to show both low affection and high conflict (‘*disharmonious’* relationship) or low affection and low conflict (‘*distant’* relationship) than parent–child relationships in Europe. Moreover, cross-national research suggests that kin ties in the U.S. are, on average, more tenuous than in many European countries (Höllinger & Haller, 1990). Consequently, even when U.S. parents maintain contact with their adult children, their bonds may not be strong and therefore less protective during a dissolution.

## Limitations and Future Directions

Our study applies a novel methodological approach to the study of depressive symptom trajectories surrounding intimate partnership dissolutions, by applying fixed-effects regression that take into account “selection” into silver splits, parent–child disconnectedness and depressive symptoms. However, our study also has limitations that invite further research. First, similar to other studies of gray divorce (Lin et al., 2024; Tosi & van den Broek, 2020), our study lacks precise information on the dissolution date. This limitation may lead to an underestimation of short-term increases in depressive symptoms following separation, as respondents may have experienced only a brief crisis and returned to their pre-separation symptom levels within a few months. Given the two-year interval between survey waves, separations that occurred well before the interview could mean we miss capturing this temporary increase.

Second, just 49 people in our sample reported parent–child disconnectedness, necessitating a cautious interpretation of our study results. We did not have sufficient statistical power to consider other sources of heterogeneity within the disconnected subgroup, such as whether a parent was disconnected from only one or multiple children, and other key characteristics of the disconnected child, including gender, whether the child was from the current or a previous union, and whether the child was biological or non-biological. Initial descriptive findings suggest that disconnectedness from a biological child may be experienced more negatively, as parents in this group reported higher average depressive symptom scores compared to those disconnected from a non-biological child (3.4 vs. 2.1 symptoms). The small sample size also precluded robust tests of heterogeneity by gender or region. Preliminary analyses suggested no significant gender differences, though disconnected women’s higher baseline symptoms may translate into greater relative risk of elevated depression. Regional patterns also pointed to larger increases in Southern Europe, but these were not statistically significant (results available from authors). Larger samples are needed to assess these differences more conclusively.

Finally, our measure of disconnectedness referred to contact during the year of the split only. We did not examine whether the disconnectedness preceded or resulted from the split, nor can we identify whether the parent or adult child instigated the disconnectedness. For instance, a strained or estranged parent–child relationship may reverberate throughout the family and ultimately destabilize the parents’ marriage. Alternatively, an adult child may recede from family interactions following a split to avoid conflict and tension or may cut off contact with the parent whom they believe was responsible for the relationship dissolution (Arránz Becker & Hank, 2022; Bowen, 1992).

Despite these limitations, our study makes important contributions to the study of gray divorce, silver splits, and late-life mental health more broadly, revealing that there may not be a single depressive symptoms profile that emerges within the context of stressful life events. Our findings suggest that researchers may benefit from using advanced longitudinal methods, such as fixed-effects regression models, to better account for selection into divorce as well as other potentially stressful family transitions in later life (Kapelle & Monden, 2024; Leopold, 2018; Tosi & van den Broek, 2020). We hope that our results offer an alternative to the narrative that later-life dissolution is uniformly distressing, and instead highlight sources of heterogeneity in mental health outcomes on the basis of one’s other family ties.

## Figures and Tables

**Fig. 3:**
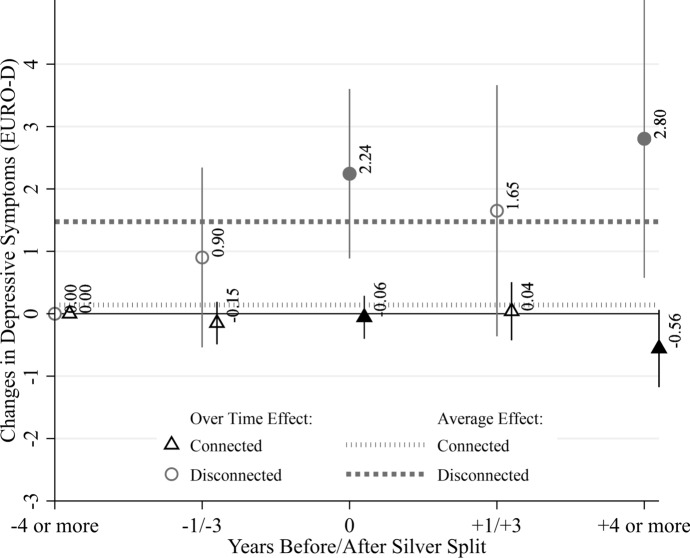
Fixed effects linear regression models predicting changes in depressive symptoms for marital dissolutions. Whiskers indicate 95% confidence intervals. Labels indicate regression coefficients. Black and grey markers indicate statistically significant differences between connected and disconnected respondents (*p* < 0.1). SHARE, waves 1, 2, 4, 5, 6, 7, 8, 9 release 9.0.0. unweighted data. Controlled for age, interview year and for imputed values.

## Data Availability

This paper uses data from SHARE Waves 1, 2, 3, 4, 5, 6, 7, 8 and 9 (DOIs: 10.6103/SHARE.w1.900, 10.6103/SHARE.w2.900, 10.6103/SHARE.w3.900, 10.6103/SHARE.w4.900, 10.6103/SHARE.w5.900, 10.6103/SHARE.w6.900, 10.6103/SHARE.w7.900, 10.6103/SHARE.w8.900, 10.6103/SHARE.w8ca.900, 10.6103/SHARE.w9.900, 10.6103/SHARE.w9ca900) see Börsch-Supan et al. (2013) for methodological details. (1) The SHARE data collection has been funded by the European Commission, DG RTD through FP5 (QLK6-CT-2001-00360), FP6 (SHARE-I3: RII-CT-2006-062193, COMPARE: CIT5-CT-2005-028857, SHARELIFE: CIT4-CT-2006-028812), FP7 (SHARE-PREP: GA No 211909, SHARE-LEAP: GA No 227822, SHARE M4: GA No 261982, DASISH: GA No 283646) and Horizon 2020 (SHARE-DEV3: GA No 676536, SHARE-COHESION: GA No 870628, SERISS: GA No 654221, SSHOC: GA No 823782, SHARE-COVID19: GA No 101015924) and by DG Employment, Social Affairs & Inclusion through VS 2015/0195, VS 2016/0135, VS 2018/0285, VS 2019/0332, VS 2020/0313 and SHARE-EUCOV: GA No 101052589 and EUCOVII: GA No 101102412. Additional funding from the German Ministry of Education and Research, the Max Planck Society for the Advancement of Science, the U.S. National Institute on Aging (U01_AG09740-13S2, P01_AG005842, P01_AG08291, P30_AG12815, R21_AG025169, Y1-AG-4553-01, IAG_BSR06-11, OGHA_04-064, BSR12-04, R01_AG052527-02, HHSN271201300071C, RAG052527A) and from various national funding sources is gratefully acknowledged (see www.share-eric.eu).

## Appendix

See Fig. 3 and Tables 5, 6, and 7.

**Table 5:** Overview of number and share of missing values

	Number	Percentage
Number of depressive symptoms	34	1.53
Silver split	0	0.00
Parent–child disconnectedness	198	8.90
Age at interview	0	0.00
Interview year	0	0.00

SHARE, waves 1, 2, 4, 5, 6, 7, 8, 9 release 9.0.0

**Table 6:** Variable composition of depressive symptoms among connected and disconnected respondents

Euro-D	Range	Mean	SD	*N*
Connected	0–12			1973
Overall		2.5	2.3	
Between			1.9	
Within			1.4	
Disconnected	0–10			209
Overall		3.6	2.5	
Between			1.8	
Within			1.8	

SHARE, waves 1, 2, 4, 5, 6, 7, 8, 9 release 9.0.0

**Table 7:** Differences in depressive symptoms following a silver split by parent–child disconnectedness (full interaction model)

	Depressive symptoms
	*B*/(SE)
Time before/since silver split (ref.: −4 years or more)	0.00
	(.)
−1/−3	0.03
	(0.17)
0 (Year of silver split)	0.09
	(0.17)
−1/−3	0.15
	(0.22)
+4 or more	−0.30
	(0.30)
Time before/since silver split (ref.: −4 years or more) × disconnected	0.00
	(.)
−1/−3 × disconnected	−0.13
	(0.56)
0 (Year of silver split) × disconnected	0.93^†^
	(0.50)
+1/+3 × disconnected	0.40
	(0.71)
+4 or more × disconnected	1.19*
	(0.53)
Person-wave observations	2216
Individual observations	546

Controlled for age, interview year and for imputed values. SHARE, waves 1, 2, 4, 5, 6, 7, 8, 9, release 9.0.0. unweighted data.

^†^*p* < 0.1, **p* < .05, ***p* < .01, ****p* < .001
